# Physicochemical and Antioxidant Properties of the Degradations of Polysaccharides from *Dendrobium officinale* and Their Suitable Molecular Weight Range on Inducing HeLa Cell Apoptosis

**DOI:** 10.1155/2019/4127360

**Published:** 2019-08-28

**Authors:** Xiaofeng Zhang, Yingyi Luo, Gang Wei, Yunrong Li, Yuechun Huang, Jiahui Huang, Chenxing Liu, Runping Huang, Guoxiong Liu, Zhaofeng Wei, Shuxiu Du

**Affiliations:** ^1^School of Pharmaceutical Sciences, Guangzhou University of Chinese Medicine, Guangzhou 510006, China; ^2^Shaoguan Institute of Danxia Dendrobium Officinale, Shaoguan 512005, China; ^3^Guangxi International Zhuang Medicine Hospital, The Affiliated International Zhuang Medicine Hospital, Guangxi Traditional Chinese Medical University, Nanning 530201, China; ^4^The First Affiliated Hospital of Guangzhou University of Chinese Medicine, Guangzhou 510405, China; ^5^Shaoguan Hejiantang Ecological Agriculture Company Ltd., Shaoguan 512000, China; ^6^Shaoguan Danxia Mountain Engineering Center of Dendrobium Technology, Shaoguan 512000, China; ^7^Shaoguan Runhu Ecological Agriculture Company Ltd., Shaoguan 512000, China

## Abstract

Different molecular weight polysaccharides of *Dendrobium officinale* (DOPs) have gradually attracted attention because of their broad biological activities. They, however, remain poorly defined whether their antitumor activity is associated with molecular weight. In this study, the physicochemical, antioxidant, and antitumor properties of DOPs, including the crude polysaccharide (DOP) and its six degradation fractions (DOP1–DOP6) extracted from *Dendrobium officinale*, were determined. Consequently, DOPs were mainly composed of different ratios of mannose and glucose as follows: 5.15 : 1, 4.62 : 1, 4.19 : 1, 4.46 : 1, 4.32 : 1, 4.29 : 1, and 4.23 : 1, and their molecular weights were significantly different ranging from 652.29 kDa to 11.10 kDa. With the concentration increase of DOPs, the scavenging capacity against OH and DPPH free radicals increased. The antitumor ability of DOPs was different that DOP1–DOP5 (Mw: 176.29 kDa–28.48 kDa) exhibited the best antiproliferation activity than DOP (Mw: 652.29 kDa) and DOP6 (Mw: 11.10 kDa) in HeLa cells rather than PC9, A549, and HepG2 cells. Moreover, it is worth mentioning that DOP1 and DOP5 showed stronger capability on inducing apoptosis of HeLa cells than DOP and DOP6 via the mitochondrial pathway by upregulating the ratio of the Bax/Bal-2 mRNA expression. The results demonstrated that DOPs can be used as the potential natural antioxidant and antitumor products in pharmaceutical industries, and the molecular weight is a crucial influential factor of their antitumor activity that 28.48 kDa–176.29 kDa is a suitable range we may refer to.

## 1. Introduction

Malignancy is one of the major causes of death, accounting for approximately 8 million deaths annually worldwide [1]. In addition, it presents the rising trend in morbidity, especially in developing countries [2]. Nevertheless, almost all chemotherapy drugs in the current market lead to serious side effects so that looking for a novel natural compound without side effects is crucial for the treatment of cancer [3].

*Dendrobium officinale* is one of the most valuable herbal plants and has been used for centuries as a herbal medicine in China and many other countries of South Asia [4]. In medicinal practice, it is used to nourish yin and enhance the production of body fluid, as well as to provide beneficial effects to the stomach and liver, moisten the lung, modulate the body's immune functions, and so on [5]. Polysaccharide is one of the main active ingredients of *Dendrobium officinale*. Several lines of evidence demonstrated that *Dendrobium officinale* polysaccharides (DOPs) exhibit anti-inflammatory activities, regulate immunity and antioxidant and antitumor activities, etc. [6, 7]. Gradually, polysaccharides in herbal medicines, including DOPs, have attracted much attention because of their antitumor activities with no significant side effects [8–10]. Furthermore, many studies have shown that the molecular weight of polysaccharides plays an important role in their biological activities [11–14]. According to reports in the literature, the lower-molecular-weight (Mw: 6.53 kDa) polysaccharide fragment from *Porphyridium cruentum* showed stronger immunoenhancing and antitumor activities [15]. Zhao et al. degraded polysaccharides from *Porphyra haitanensis* using the H_2_O_2_ system to obtain fragments of different molecular weights and proved that the antioxidant activity of them was closely related to the molecular weight [16]. However, in terms of DOPs, some studies applied polysaccharides by fractional precipitation or the total polysaccharides to investigate the antitumor activity [17, 18], and fewer reports have published concerning polysaccharides that degraded into a series of molecular weights but lacking comparison of molecular weights [18].

In this paper, we extracted and compared the antioxidant and antitumor activities in vitro of seven polysaccharide fractions from *Dendrobium officinale* with different molecular weights, providing the natural novel antioxidant and antitumor compounds, as well as a molecular weight reference for polysaccharides on antitumor activity in other medicinal materials.

## 2. Materials and Methods

### 2.1. Plant Materials

The artificially cultivated stems of *Dendrobium officinale* Kimura et Migo were collected from Yunnan Province in China and identified by Prof. Gang Wei, Guangzhou University of Chinese Medicine, Guangdong, China. These fresh samples were cut into segments (0.5 cm) and ground into powder after drying in an oven at 60°C.

### 2.2. Reagents and Cell Culture

The reagents used in extraction and degradation were 1-phenyl-3-methyl-5-pyrazolone (PMP) (Macklin, Shanghai, China); monosaccharide standards of galactose (Gal), mannose (Man), glucose (Glu), and rhamnose monohydrate (Rha) (National Institutes for Food and Drug Control, Beijing, China); and T-series dextran (Mw: 1270, 5220, 11600, 48600, 80900, 273000, and 409800 Da) (Sigma-Aldrich, St. Louis, MO, USA). The reagents used in the experiment in vitro were salicylic acid (Zhiyuan Chemical Reagent Co., Ltd., Tianjin, China); 1,1-diphenyl-2-picrylhydrazyl (DPPH), butylated hydroxytoluene (BHT), and 3-(4,5-dimethylthiazol-2-yl)-2,5-diphenyl tetrazolium bromide (MTT) (Sigma-Aldrich); 1640 medium, Dulbecco's modified Eagle's medium (DMEM), fetal bovine serum (FBS), phosphate buffer saline (PBS), and trypsin (Gibco, Thermo Fisher Scientific™, Suzhou, China); Eagle's minimal essential medium (MEM) and Annexin V-FITC/PI cell apoptosis array kit (KeyGene, Jiangsu, China); PrimeScript RT Reagent Kit with gDNA Eraser and SYBR® Premix DimerEraser™ (Takara, Otsu, Shiga, Japan); and TRIzol reagent (Life Technologies, Carlsbad, USA). All other reagents were of analytical grade.

The PC9, A549, and HeLa cell lines were purchased from KeyGEN BioTECH Corp., Ltd., USA, and HepG2 cell lines were purchased from Jennio Biotech Co., Ltd., GuangZhou, China. All cells were cultured according to the manufacturer's instruction that PC9 and A549 cells were cultured in the 1640 medium, HepG2 cells were maintained in DMEM, while HeLa cells were cultured in the MEM. All types of media contained 10% (v/v) FBS, 100 U/ml penicillin, and 100 *μ*g/ml streptomycin with a humidified atmosphere containing 5% CO_2_ at 37°C.

### 2.3. Extraction and Degradation of DOPs with Different Molecular Weights

The crude polysaccharide of *Dendrobium officinale* (DOP) was extracted as described previously [19]. In brief, 50 g of *Dendrobium officinale* powder was refluxed with 500 mL of petroleum ether at 70°C for 2 h to remove lipids. After the removal of the supernatant by filtration, the residue was dried at room temperature and extracted with 500 mL of 80% ethanol at 90°C for 2 h to discard small molecule compounds. The filtration residue was collected and extracted with 1500 mL of distilled water three times at 100°C for 2 h. All the precipitates were combined and centrifuged at 4000 rpm for 10 min. The supernatant was then collected and concentrated. Four times the volume of ethanol was slowly added and lyophilized at 4°C overnight to obtain the polysaccharide. The protein in the polysaccharides was removed using the Sevage method [20] three to five times until no white floccules were evident at the border between the water and chloroform to obtain DOP.

The degradation of DOP with different molecular weights was evaluated by the method our group used for the long term [18] but with slight differences. 3.6 g of DOP was dissolved in 314.7 mL of distilled water in an ultrasonic bath. After dissolving Vitamin C (Vc), iron chloride tetrahydrate (FeCl_2_·4H_2_O), and 30% H_2_O_2_ with distilled water, six 50 mL conical flasks of the reaction solution were prepared according to the method in Table 1. The mixture was shaken and reacted in the dark for 2 h at room temperature (25°C). The reaction product was transferred to a 300 Da dialysis bag and dialysed for 3 days against flowing distilled water. Followed by centrifugation at 6000 rpm for 10 min, the supernatant was concentrated and freeze-dried. Six different polysaccharide fragments (DOP1–DOP6) were obtained.

### 2.4. Physicochemical Property Analysis

#### 2.4.1. Determination of the Polysaccharide Content

The polysaccharide content was determined using the phenol-sulfuric acid method [21] taking the glucose as the standard sample. The concentration was measured using the standard curve based on the anhydrous glucose standard references.

#### 2.4.2. Monosaccharide Composition Analysis

The polysaccharide sample DOPs were dissolved with distilled water to the concentration of 1 mg/mL. And the pH of the samples was adjusted to 7.0 using NaOH solution after the 3 M HCl was added to the sample solution, while the concentration of mixed monosaccharide standards, galactose (Gal), mannose (Man), glucose (Glu), and rhamnose monohydrate (Rha), was adjusted to 0.45 mg/mL. The solutions of samples and mixed monosaccharide standards were converted to their derivatives with 0.5 M PMP. All the solutions were allowed to react at 70°C in an oven for 100 min and then cooled to ambient temperature (25°C) and added with 0.3 M HCl. Last, chloroform was added and mixed via vortexing, followed by centrifugation at 3000 rpm for 10 min to discard chloroform. This procedure was repeated 3 times until no colour was evident in the chloroform layer. The lower water-phase layers were the sample solution and mixed monosaccharide standard solution.

The monosaccharide compositions of DOPs were analysed by high-performance liquid chromatography (HPLC) (Shimadzu, Kyoto, Japan) equipped with a Kromasil 100-5-C18 column (4.6 × 250 mm, 5 *μ*m) (Akzo Nobel, Sweden). The mobile phase was composed of acetonitrile (solvent A) and 0.1 M phosphate buffer (solvent B) at a ratio of 15.5 : 84.5 under isocratic elution, and the flow rate was 0.8 mL/min.

#### 2.4.3. Detection of the Molecular Weight

The concentration of DOP samples was adjusted to 2 mg/mL with distilled water to obtain the sample solutions. T-series dextrans (Mw: 1270, 5220, 11600, 48600, 80900, 273000, and 409800 Da) were used as standard references. High-performance gel permeation chromatography (HPGPC) was applied in the experiment. After the standard curve was drawn, the molecular weight and purity of the DOPs were determined by an Agilent 1100 series HPLC system (Palo Alto, CA, USA) equipped with an RI-101SHDEX RID detector (Tosoh, Tokyo, Japan). The mobile phase was 20 mM CH_3_COONH_4_ with a flow rate of 0.8 mL/min.

### 2.5. Analysis of Antioxidant Activities

#### 2.5.1. 1,1-Diphenyl-2-picrylhydrazyl (DPPH) Radical-Scavenging Assay

The free-radical-scavenging capacity of DOPs was analysed using DPPH according to a previously reported method with slight modifications [22]. Generally, DPPH was dissolved with methanol in an ultrasonic bath, followed by shaking vigorously and storage away from light. 150 *μ*L of DOP solutions at different concentrations (0.5, 1.0, 2.0, 3.0, and 5.0 mg/mL) was fixed by 50 *μ*L of DPPH solution in the 96-well plates and kept at room temperature for 30 min under lightproof condition. Thereafter, the reaction was stopped by cooling at room temperature, and the absorption at 510 nm was determined by an iMark microplate absorbance reader (Bio-Rad Inc., CA, USA), while the solution of butylated hydroxytoluene (BHT) was used as a positive control. The test was performed in triplicate, and the DPPH radical-scavenging rate was calculated as follows:

(1) $$\begin{array}{c} \text{DPPH radical}‐\text{scavenging rate}\left(\%\right)=\left[1-\frac{\left(A_{1}-A_{2}\right)}{A_{0}}\right]\;\times 100\%, \end{array}$$

where *A*_1_ refers to the absorption of DOP samples with DPPH solution, *A*_2_ refers to the absorption of DOP samples without DPPH solution, and *A*_0_ refers to the absorption of control (DPPH solution without DOP samples).

#### 2.5.2. Hydroxyl (OH) Radical-Scavenging Assay

OH radical-scavenging activity was measured according to the method described but with slight innovation [23]. In brief, 2 mL of DOP solutions at different concentrations (0.5, 1.0, 2.0, 3.0, and 5.0 mg/mL) was transferred to a test tube, and 2 mL of 6 mM·FeSO_4_ solution and 2.4 mM·H_2_O_2_ solution was added to the test tube for 10 min. Thereafter, 2 mL of 6 mM salicylate was added and incubated at 37°C for 30 min. The mixture was centrifuged at 4000 rpm for 10 min, and the supernatant was applied to measure the absorption at 510 nm using an iMark microplate absorbance reader. The solution of Vc was used as a positive control, and the hydroxyl radical-scavenging rate was expressed as follows:

(2) $$\begin{array}{c} \text{OH radical}‐\text{scavenging rate}\left(\%\right)\;=\;\left[1-\frac{\left(A_{i}-\;A_{j}\right)}{A_{0}}\right]\;\times 100\%, \end{array}$$

where *A*_*i*_ refers to the absorption of the DOP samples with salicylate, *A*_*j*_ refers to the background absorption of the DOP samples using distilled water to replace salicylate, and *A*_0_ refers to the blank absorption using distilled water to replace DOP samples.

### 2.6. Antitumor Activity Analysis

#### 2.6.1. Growth Inhibition Activity

The growth inhibition activity of DOPs in PC9 cells, A549 cells, HepG2 cells, and HeLa cells was measured using the MTT-based colorimetric method. The cells in the logarithmic phase were seeded at a density of 3 × 10^4^ cells/well in 96-well plates and incubated at 37°C in 5% CO_2_. 24 h later, the medium was removed, and 100 *μ*L of DOP solutions with concentrations of 25, 50, 100, 200, and 400 *μ*g/mL in the fresh FBS-free medium was added to HepG2 cells and HeLa cells. While evaluated by the preexercise, there existed little difference about the concentration of DOPs in PC9 cells and A549 cells that 50, 100, 200, 400, and 800 *μ*g/mL were added to incubate cells. The FBS-free medium was used as the blank control, and five biological repeats were performed in each experiment. After incubation for 24 h, 20 *μ*L of the MTT reagent (5.0 mg/mL) was added to further incubate for 4 h for producing the purple formazan crystals. Then, the medium was removed carefully, and 100 *μ*L of DMSO was added to each well. The absorbance at a wavelength of 490 nm was measured using an iMark microplate absorbance reader. And the cell viability rate was calculated according to the following formula:

(3) $$\begin{array}{c} \text{cell viability rate}\left(\%\right)\;=\;\frac{A_{1}}{A_{0}}\;\times \;100\%, \end{array}$$

where *A*_1_ and *A*_0_ represent the absorbance of the DOP sample and the blank control, respectively.

#### 2.6.2. Flow Cytometric Analysis of Cell Apoptosis

HeLa cells were seeded in 6-well plates (2 × 10^4^ cells/well) and incubated at 37°C in 5% CO_2_ for 24 h. The medium was removed, and DOP, DOP1, DOP5, and DOP6 were added at a concentration of 100 *μ*g/mL for 24 h. According to the manufacturer's instructions, the HeLa cells digested with trypsin (non-EDTA) were collected into a centrifuge tube and centrifuged at 1000 rpm for 5 min. The supernatant was removed, and the cells were washed with 800 *μ*L of PBS 3 times. 500 *μ*L of the binding buffer was added to resuspend the cells, followed by staining with 5 *μ*L of Annexin V-FITC and PI. After reaction of the solution for 10 min at room temperature in the dark, the samples were immediately analysed using a FACSCanto II flow cytometer (BD Biosciences, USA).

#### 2.6.3. Real-Time Quantitative PCR (RT-qPCR) Assay

HeLa cells were seeded in 6-well plates (1 × 10^5^ cells/well) and incubated in a CO_2_ incubator. 24 h later, the cells were exposed to DOP, DOP1, DOP5, and DOP6 with a concentration of 100 *μ*g/mL for another 24 h. According to the manufacturer's protocol, the total RNA was extracted using the TRIzol reagent. After gDNA was discarded using a PrimeScript RT Reagent Kit with gDNA Eraser, SYBR® Premix DimerEraser™ was used to convert the total RNA for synthesizing cDNA. The expression of Bax and Bcl-2 mRNA relating to cell apoptosis was detected using a StepOnePlus™ Real-Time PCR instrument (Applied Biosystems Inc., USA). The primer *β*-actin (Table 2) was used as an internal reference to normalize the variability in the expression levels. The target gene expression levels were calculated using the 2^−ΔΔCt^ method.

### 2.7. Data Analysis

All statistical data were expressed as mean ± SD, and the results from each group were obtained using triplicate samples independently using the Prism 5 software package (GraphPad Software Inc., La Jolla, CA, USA). Statistical differences among the groups were assessed using a one-way ANOVA, and Fisher's least significant difference (LSD) test was used to determine significant differences for different samples. ^*∗*^*P* < 0.05 was considered to be statistically significant, while ^*∗∗*^*P* < 0.01 was considered to be highly statistically significant.

## 3. Results

### 3.1. Physicochemical Properties of DOPs

**Figure 1:**
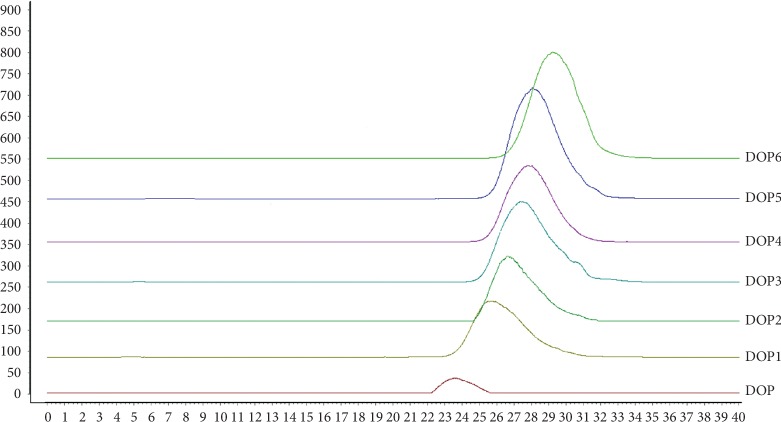
HPLC of molecular weight of polysaccharides of *Dendrobium officinale* (DOPs).

The crude polysaccharide of *Dendrobium officinale* named “DOP” was obtained through water extraction and alcohol precipitation as well as deproteinization. Hydrogen peroxide is an ideal polysaccharide depolymerization reagent. The polysaccharide degradation reaction induced by hydrogen peroxide is moderate, and the extent of degradation can be controlled easily by changing the degradation reaction conditions; consequently, different polysaccharide degradation degrees can be obtained [24]. Thus, DOP was degraded through six different concentrations of the H_2_O_2_-Vc-Fe^2+^ system to obtain six polysaccharide fractions, namely, DOP1, DOP2, DOP3, DOP4, DOP5, and DOP6 (DOP1–DOP6). The yields of DOP and DOP1–DOP6 (DOPs) were calculated to be 18.89%, 68.51%, 75.98%, 76.54%, 67.43%, 74.11%, and 68.95%, respectively. The total polysaccharide contents of DOPs were determined by the phenol-sulfuric acid method to be 62.61%, 73.97%, 76.81%, 79.46%, 78.30%, 78.12%, and 89.98%, respectively. As depicted in Table 3, DOPs have different molecular weights ranging from 11.10 kDa to 652.29 kDa. The HPLC maps of DOPs are shown in Figure 1, and the purity of the DOPs were greater than 98% as determined by HPLC.

**Figure 2:**
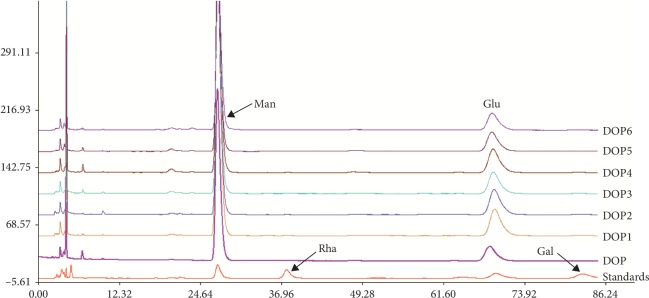
HPLC of monosaccharide composition of the mixed monosaccharide standards and DOPs. Man: mannose; Rha: rhamnose monohydrate; Glu: glucose; Gal: galactose.

The monosaccharide composition of DOPs could be determined after comparing the HPLC results of DOP samples with those of monosaccharide standards. As shown in Figure 2, all DOP samples mainly consisted of mannose and glucose. In addition, the content of mannose was higher than that of glucose in all DOP samples. The mannose-to-glucose ratio of DOPs can be obtained by calculating the ratio of the peak areas as follows: 5.15 : 1, 4.62 : 1, 4.19 : 1, 4.46 : 1, 4.32 : 1, 4.29 : 1, and 4.23 : 1, respectively. As shown in Table 3, the relative content of mannose in DOPs was decreased after degradation.

### 3.2. Antioxidant Activity of DOPs In Vitro

Polysaccharides extracted from *Dendrobium Sw.* and some other plants exhibited significant antioxidant activity in vitro, including scavenging abilities on DPPH, OH, ABTS^+^, and Fe^2+^ radicals [25, 26].

**Figure 3:**
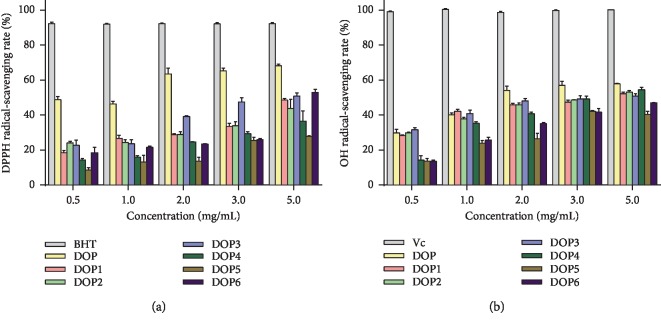
DPPH (a) and OH (b) radical-scavenging activity of DOPs (0.5–5.0 mg/mL).

DPPH is a free-radical compound that has been widely used to evaluate the free-radical-scavenging ability of natural materials. In this study, the antioxidant activities of DOPs were compared. The scavenging ability of DOPs on DPPH radicals is shown in Figure 3(a), compared with BHT as a positive standard. At the concentrations of 0.5–5.0 mg/mL, the DPPH radical-scavenging rates of DOPs and BHT were in the range of 48.8%–68.1%, 18.5%–48.6%, 24.0%–43.8%, 22.7%–50.8%, 14.3%–36.5%, 8.6%–27.9%, 18.5%–52.9%, and 91.9%–92.2%, respectively. Furthermore, when comparing with DOP1–DOP6 with lower molecular weights, DOP with a higher molecular weight of 652.29 kDa showed the best DPPH radical-scavenging ability gradually close to that of BHT. The results indicated that all DOPs exerted effective DPPH radical-scavenging ability in a dose-dependent manner and be influenced by molecular weight.

OH radical is regarded to be the most harmful reactive oxygen species and cause severe damage to adjacent biomolecules, resulting in cancer in vivo. The scavenging ability of DOPs against OH radicals is shown in Figure 3(b). In the concentration range of 0.5–5.0 mg/mL, the scavenging rate of DOPs and Vc, used as the positive standard, was 29.7%–57.8%, 28.3%–52.1%, 29.8%–53.1%, 31.5%–50.8%, 14.2%–54.4%, 13.7%–47.0%, 13.5%–47.0%, and 99.0%–100.3%, respectively. Similar to the DPPH radical-scavenging assay, the antioxidant activity of DOP with a molecular weight of 652.29 kDa against OH radicals was obviously higher than that of DOP4–DOP6 with smaller molecular weights in a dose-dependent manner, and the results were the same when comparing DOP1–DOP3 to DOP4–DOP6. The results indicated that the OH radical-scavenging abilities of DOPs have a certain relationship with their molecular weights, and DOPs may be beneficial to protect from oxidative damage.

Above all, DOPs exhibited noticeable antioxidant activity against DPPH and OH radicals in a dose-dependent manner, and molecular weight was one of the influence factors.

### 3.3. Antitumor Activity of DOPs In Vitro

#### 3.3.1. Growth Inhibitory Ability of DOPs in Four Kinds of Tumor Cells

**Figure 4:**
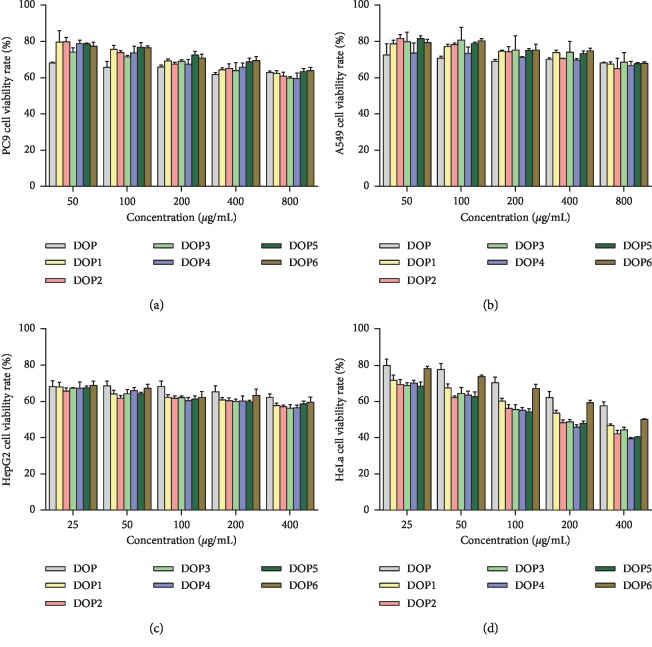
Growth inhibition activity of DOPs on tumor cells for 24 h. (a) PC9 cell viability rate treated with 50–800 *μ*g/mL of DOPs. (b) A549 cells treated with 50–800 *μ*g/mL of DOPs. (c) HepG2 cells treated with 25–400 *μ*g/mL of DOPs. (d) HeLa cells treated with 25–400 *μ*g/mL of DOPs.

In this study, the growth inhibition activity of DOPs on cancer cells PC9, A549, HepG2, and HeLa was tested by the MTT assay. As shown in Figures 4(a)–4(d), DOPs displayed different extents of growth inhibition activity in four kinds of tumor cells. At the highest concentration screened by the preexperiment, the cell viability rates of DOPs (800 *μ*g/mL) on PC9 cells were 62.9%, 62.4%, 60.9%, 59.8%, 59.6%, 63.4%, and 63.9%, respectively; the cell viability rates on A549 cells (800 *μ*g/mL) were 68.2%, 67.5%, 65.0%, 68.5%, 66.7%, 67.7%, and 67.9%, respectively; the minimum cell viability rates on HepG2 cells (400 *μ*g/mL) were 62.3%, 57.7%, 57.1%, 56.3%, 56.5%, 58.6%, and 59.6%, respectively; and the minimum cell viability rates on HeLa cells (400 *μ*g/mL) were 57.7%, 46.7%, 42.2%, 44.4%, 39.4%, 40.4%, and 50.0%, respectively. Analysing about the data, the growth inhibition activity of DOPs on PC9, A549, and HepG2 cells was found to be weaker when compared to HeLa cells, especially on PC9 and A549 cells, and the difference of the growth inhibition activities among DOPs was not obvious. Furthermore, the dose dependence of DOPs on HeLa cells was most evident, and the effect of DOP1–DOP5 (Mw: 28.48–176.29 kDa) on the growth inhibition of HeLa cells was more effective than that of DOP (Mw: 652.29 kDa) and DOP6 (Mw: 11.10 kDa) in the concentration range of 25–400 *μ*g/mL. From the results shown, it could be concluded that DOPs, especially DOP1–DOP5, had a better growth inhibition activity on HeLa cells within these cancer calls evaluated in this experiment. Thus, HeLa cells and DOP, DOP1, DOP5, and DOP6 were selected to be performed in the subsequent experiments for exploring the relationship between different molecular weights of DOPs and their antitumor activity.

#### 3.3.2. Cell Apoptosis Analysis of DOP, DOP1, DOP5, and DOP6 in HeLa Cells

**Figure 5:**
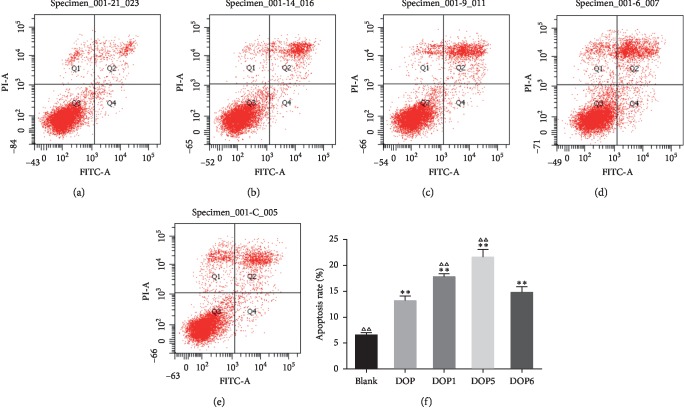
Apoptosis rate of HeLa cells induced by (a) blank, (b) DOP, (c) DOP1, (d) DOP5, and (e) DOP6 for 24 h, as well as (f) the column bar graph of apoptotic HeLa cells. ^*∗*^*P* < 0.05 and ^*∗∗*^*P* < 0.01 compared with the blank group. ^ΔΔ^*P* < 0.01 compared with the DOP group.

The antitumor activities of DOP, DOP1, DOP5, and DOP6, measured by the spontaneous apoptosis rate of HeLa cells, were tested using the Annexin V and PI double-staining assay with flow cytometry. As depicted in Figure 5(a), the apoptosis rate of the blank group was 6.6%. After incubation with DOP, DOP1, DOP5, and DOP6 (100 *μ*g/mL) for 24 h, the apoptosis rates were increased to 13.2%, 17.8%, 21.6%, and 14.8% (Figures 5(b)–5(e)), respectively. The values enabled the conclusion that DOP, DOP1, DOP5, and DOP6 could significantly induce cell apoptosis (*P* < 0.01). Meanwhile, the apoptosis rates of DOP1 and DOP5 were obviously higher than those of DOP and DOP6, suggesting that the apoptosis ability of DOPs might be influenced by the molecular weight similar to the results of the MTT assay, and 28.48 kDa–176.29 kDa is a suitable molecular weight range of DOPs on inducing apoptosis capability in HeLa cells.

#### 3.3.3. Expression of Bcl-2 and Bax Treated with DOP, DOP1, DOP5, and DOP6 in HeLa Cells

**Figure 6:**
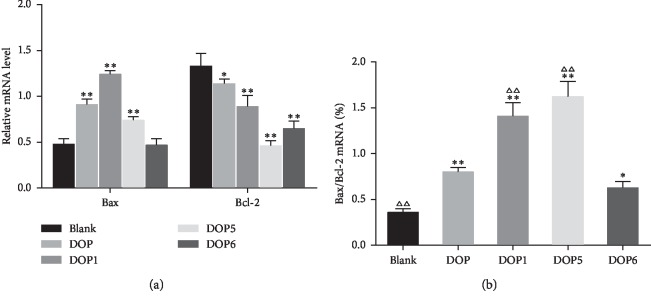
(a) Relative expression of Bax and Bcl-2 mRNA and (b) the ratio of them in HeLa cells induced by blank, DOP, DOP1, DOP5, and DOP6 for 24 h. ^*∗*^*P* < 0.05 and ^*∗∗*^*P* < 0.01 compared with the blank group. ^ΔΔ^*P* < 0.01 compared with the DOP group.

To further explore the relationship between DOPs with different molecular weights and antitumor activity, the RT-qPCR assay was applied to assess the expression level changes of the antiapoptotic gene Bcl-2 and proapoptotic gene Bax after induction by DOP, DOP1, DOP5, and DOP6 (100 *μ*g/mL) for 24 h. Compared with the blank group (Figure 6(a)), the expression level of Bcl-2 and Bax mRNA in HeLa cells was downregulated and upregulated, respectively. The ratio of Bax/Bcl-2 is an index to indicate apoptosis regulation in cells, and the effect of inducing apoptosis would be enhanced as this ratio increased. In this study, the ratio of Bax/Bcl-2 in HeLa cells (Figure 6(b)) was increased after the induction of DOP, DOP1, DOP5, and DOP6. In addition, after mathematical analysis, this ratio was related to the molecular weight of the polysaccharide fractions. When we compared DOP1 and DOP5 groups with DOP and DOP6 groups, the ratio of Bax/Bcl-2 mRNA expression was higher, suggesting that DOP1 (Mw: 176.29 kDa) and DOP5 (Mw: 28.48 kDa) showed a better effect than DOP (Mw: 652.29 kDa) and DOP6 (Mw: 11.10 kDa).

These findings suggested that DOP1 and DOP5 might induce apoptosis of HeLa cells by upregulating the ratio of Bax/Bcl-2 mRNA expression via the mitochondrial pathway, confirming the result again that DOPs with molecular weights ranging from 28.48 kDa to 176.29 kDa had a stronger ability to induce apoptosis of HeLa cells.

## 4. Discussion

Previous studies have proven that *Dendrobium officinale* has broad bioactivities, including immunomodulation, antifatigue, antitumor, antioxidation, digestion promotion, stimulation of salivary secretion, hypoglycemic, and antihypertension [27]. However, as one of the major active components in *Dendrobium officinale*, few reports have described the antitumor effect of DOPs, particularly the comparison of different molecular weights of polysaccharides. In addition, accumulating evidence suggested that the bioactivity of the polysaccharide is related to its molecular weight, viscosity, solubility, and primary and fine structures [28, 29]. Hence, we obtained seven polysaccharide fractions with significantly different molecular weights ranging from 11.10 kDa to 652.29 kDa from *Dendrobium officinale* after degradation by the Sevage method. In this study, DOPs exhibited good antioxidant ability on scavenging the DPPH and OH free radicals and expressed varying degrees of antitumor activity in PC9, A549, HepG2, and HeLa cells. Among these cells, in the MTT assay, DOP1–DOP5 (Mw: 28.48 kDa–176.29 kDa) possessed the best antiproliferation activities than DOP (Mw: 652.29 kDa) and DOP6 (Mw: 11.10 kDa) in HeLa cells. Given the results, we speculated that DOPs with a molecular weight ranging from 28.48 kDa to 176.29 kDa had the better antitumor activity in HeLa cells. Thus, we evaluated the apoptosis rate induced by DOP, DOP1, DOP5, and DOP6 in HeLa cells via the apoptosis assay. The results suggested that DOP1 and DOP5 can obviously induce the apoptosis of HeLa cells than DOP and DOP6, consistent with the results of the MTT assay. To further explore the antitumor mechanism, we studied the relative mRNA expression in HeLa cells by the RT-qPCR assay. Consequently, compared with DOP and DOP6, DOP1 and DOP5 could better downregulate the expression of Bcl-2 mRNA and upregulate the expression of Bax mRNA, resulting in cell apoptosis, consistent with the results above again. Therefore, we suggest that DOPs with a molecular weight range of 28.48 kDa–176.29 kDa exhibit a significant antitumor activity through the mitochondrial pathway.

Molecular weight is one of the important indexes for the quality control of polysaccharide products and medicines. It will be difficult for polysaccharide with a very high molecular weight to be transferred to the membrane in vivo, consequently preventing the polysaccharide from exerting its bioactivity. In our study, DOP (Mw: 652.29 kDa) showed no significant antitumor activities. It is reasonable to consider that this result may be because of high molecular weight. By contrast, polysaccharides with a lower molecular weight will be easy to bind on the bioactive binding sites. However, it will be inappropriate if the molecular weight is too low because it would be impossible to form the aggregate structure for an effect, which we can refer to DOP6 (Mw: 11.10 kDa). Thus, an appropriate molecular weight is a critical factor for the biological activity of the polysaccharide. For example, a polysaccharide DOPA-1 from *Dendrobium officinale* in Huoshan has an average molecular weight of 229 kDa close to the upper limit of our suitable molecular weight range (176.29 kDa), resulting in induction of apoptosis in tumor cells through altered mitochondrial function, ROS production, and altered apoptosis-related protein expression [30]. Similarly, the polysaccharide DCPP1a-1 isolated from the protocorms of *Dendrobium officinale* with a molecular weight of 189 kDa showed significant antitumor effects on H22-bearing mice and significantly raised the thymus and spleen indexes [31]. Moreover, we speculated that the molecular weight of DOPA-1 and DCPP1a-1 may decrease closer to the lower limit of our suitable molecular weight range (28.48 kDa) after degradation by the gastrointestinal in vivo. The polysaccharide DOP-70 (Mw: 50.3 kDa, within our suitable molecular weight range) from *Dendrobium officinale*, obtained by our group before [17], also exhibited a better anti-HepG2 cell activity in vitro. In addition, through preexperiments, we have investigated that the antitumor activity of DOPs isolated by our group before (Mw: 776 kDa) [32] and monosaccharide (Mw < 10 kDa), whose molecular weight was all outside our suitable molecular weight range, was not significant. Thus, we consider that the antitumor activity of DOPs is related to their molecular weights, and 28.48 kDa–176.29 kDa is a suitable molecular weight range. However, the structure analysis of DOPs has not yet been done in this experiment, and whether there is a link between different polysaccharide structures and their antitumor activities needs to be further studied.

## 5. Conclusions

In summary, our results provide the promising evidence that molecular weight plays a critical role in the antitumor activity of DOPs isolated from *Dendrobium officinale* and initially find a suitable weight range for the first time. Notably, DOPs displayed a good antioxidant activity against DPPH and OH free radicals, as well as the antitumor activity on growth inhibition in several tumor cells. However, DOP1 and DOP5 had the better significant capability of inducting apoptosis and upregulating the ratio of Bax/Bcl-2 expression in HeLa cells owning to different molecular weights. To conclude, polysaccharides from *Dendrobium officinale*, with a molecular weight range of 28.48 kDa–176.29 kDa, could be better used as the natural novel antioxidant and antitumor compounds, providing the antitumor activity of polysaccharides from other medicinal materials as a reference of molecular weight.

## Figures and Tables

**Table 1:** Degradation method of DOP.

Sample	100 mM Vc (mL)	3% H_2_O_2_ (mL)	1000 mM FeCl_2_·4H_2_O (mL)	DOP (mL)	Water (mL)
1	0.5	0.05	0.05	43.7	5.4
2	1	0.1	0.1	43.7	4.8
3	2	0.2	0.2	43.7	3.8
4	4	0.4	0.4	43.7	1.2
5	5	0.5	0.5	43.7	0.3
6	6	0.6	0.6	43.7	0

**Table 2:** Primers used for RT-qPCR.

Gene	Forward primer	Reverse primer
*β*-Actin	5′-AGCACAGAGCCTCGCCTTT-3′	5′-ATCACGCCCTGGTGCCT-3′
Bax	5′-TCATGGGCTGGACATTGGAC-3′	5′-GAGACAGGGACATCAGTCGC-3′
Bcl-2	5′-TTCTTTGAGTTCGGTGGGGTC-3′	5′-TGCATATTTGTTTGGGGCAGG-3′

**Table 3:** Chemical characteristics of DOPs.

Samples	Yield (%)	Total sugar (%)	Mw (kDa)	Peak value ratio (Man : Glu)
DOP	18.89	62.61	652.29	5.15 : 1.00
DOP1	68.51	73.97	176.29	4.62 : 1.00
DOP2	75.98	76.81	75.92	4.19 : 1.00
DOP3	76.54	79.46	40.96	4.46 : 1.00
DOP4	67.43	78.30	33.10	4.32 : 1.00
DOP5	74.11	78.12	28.48	4.29 : 1.00
DOP6	68.95	89.98	11.10	4.23 : 1.00

Mw: molecular weight; Man: mannose; Glu: glucose.

## Data Availability

The data used to support the findings of this study are available from the corresponding author upon request.
